# Interpreting the orientation of objects: A cross-disciplinary review

**DOI:** 10.3758/s13423-024-02458-8

**Published:** 2024-02-01

**Authors:** Irina M. Harris

**Affiliations:** School of Psychology, University of Sydney, Brennan MacCallum Building A18, Sydney, NSW 2006 Australia

**Keywords:** Object orientation, Object recognition, Orientation agnosia, RSVP, Repetition blindness, Attentional blink

## Abstract

Is object orientation an inherent aspect of the shape of the object or is it represented separately and bound to the object shape in a similar way to other features, such as colour? This review brings together findings from neuropsychological studies of patients with *agnosia for object orientation* and experimental studies of object perception in healthy individuals that provide converging evidence of separate processing of object identity and orientation. Individuals with agnosia for object orientation, which typically results from damage to the right parietal lobe, can recognize objects presented in a range of orientations yet are unable to interpret or discriminate the objects’ orientation. Healthy individuals tested with briefly presented objects demonstrate a similar dissociation: object identity is extracted rapidly in an orientation-invariant way, whereas processing the object’s orientation is slower, requires attention and is influenced by the degree of departure from the canonical orientation. This asymmetry in processing can sometimes lead to incorrect bindings between the identity and orientation of objects presented in close temporal proximity. Overall, the available evidence indicates that object recognition is achieved in a largely orientation-invariant manner and that interpreting the object’s orientation requires an additional step of mapping this orientation-invariant representation to a spatial reference frame.

We can identify objects in a fraction of a second, despite enormous variability in their appearance. Consider recognizing an object as a “dog” (what is termed “basic-level” recognition)—we can do that without any effort, regardless of whether it is near or far (and, therefore, projects a different image on the retina), whether it is our dog or the neighbor’s, whether we see it as a three-dimensional “real” dog, as a black-and-white photograph, a stylized line drawing, or a mere shadow. Clearly, our visual system is equipped with mechanisms that are able to disregard such variations in surface information and infer the structure and identity of the object with apparently little cost to performance or change in patterns of brain activation (Kourtzi & Kanwisher, 2000). Another aspect that our visual system is able to compensate for is variation in the orientation of the object, although changes in orientation do often incur a cost. In general, it takes longer to identify objects when they are rotated away from their usual, or most familiar, orientation and these changes are often proportional to the degree of misorientation of the object (Jolicoeur, 1985; Jolicoeur & Milliken, 1989; McMullen & Jolicoeur, 1990; Tarr & Pinker, 1989). Such findings have prompted a number of researchers to suggest that our visual system represents objects as they appear to the observer from specific viewpoints and the process of recognition involves matching these views to a representation of the object stored in memory through some form of spatial transformation.

Such *viewpoint-dependent* theories of object recognition differ with respect to the exact nature of the stored representations and the processes involved in matching the visual input to the stored memory representations. Some theorists believe that memory representations consist of a single image, corresponding to the most familiar view—termed the *canonical view* (Palmer et al., 1981; Rock, 1973). Others argue that multiple views are stored, corresponding to different instances encountered during one’s experience with the object (Tarr, 1995; Tarr & Pinker, 1989). Each type of model has implications for the kinds of transformations involved, with the former requiring that novel views of objects be recognized by transforming the input image to the stored canonical representation and the latter by transformation to the nearest stored view. One popular mechanism that has been proposed to underly the transformation is mental rotation—that is, a rigid analog transformation through the intervening angles between the depicted view and the stored representation (Jolicoeur, 1985, 1990; Tarr, 1995; Tarr & Bülthoff, 1995)—although, more recent evidence argues against such mechanism being required for object recognition (Gauthier et al., 2002; Harris et al., 2002; Hayward et al., 2006). Alternative mechanisms include interpolation between previously stored views (Bülthoff & Edelman, 1992; Edelman, 1999) or the alignment of key features in the image with those of stored representations (Ullman, 1989). Despite these differences, all viewpoint-dependent theories of recognition argue that recognition is mediated by image-based representations of objects from specific viewpoints, which inherently code the orientation of the object relative to the observer.

In contrast, *view-invariant* theories of object recognition postulate that objects are represented on the basis of distinctive features and their interrelations, which remain constant across changes in viewpoint (Biederman, 1987; Marr, 1982; Marr & Nishihara, 1978). Marr proposed a hierarchical theory of recognition, in which the initial steps towards recognition are viewpoint-dependent (the *primal sketch*, which provides low-level information about edges and vertices present in the image and the *2½D sketch*, which provides information about surfaces as defined from a viewer’s perspective). However, he did not believe that this was sufficient to support recognition and suggested that recognition required the construction of a 3D object representation in which the object features are defined with respect to a reference frame centered on the object (defined by the object’s principal axes), rather than the viewer. Therefore, although Marr’s theory is generally regarded as espousing viewpoint-invariant object recognition, in practice it is only partially view invariant. This is because a viewpoint-dependent representation is a necessary step in generating a fully viewpoint-invariant object-centered representation, but once that object-centered representation is constructed, recognition should be possible irrespective of viewpoint or orientation. Corballis (1988) proposed a modification of Marr’s theory in which recognition could proceed largely independently of any reference frame, based on the initial identification of salient features. This is a relatively crude process, but Corballis argued that it was sufficient to activate a stored representation of the object in memory, which contained richer information about the object. This would include information about the object’s internal reference frame and how features are organized with respect to this, as well as information about the usual orientation of the object which could be used to refine the initially crude recognition and guide any spatial normalization required to recognize misoriented objects. Therefore, in Corballis’ view, recognition can be achieved before a reference frame is assigned to the object, implying that the activation of an object’s representation in memory may bypass a viewer-centered or viewpoint-dependent representation.

Another object-centered theory which gives primacy to component features was proposed by Biederman (1987). According to this theory, objects are represented as structural descriptions specifying the spatial relations among viewpoint-invariant volumetric primitives called “geons,” with a relatively small number of geons (36) providing the building blocks for all possible objects. The geon-structural-description (GSD) theory states that as long as two views of an object activate the same structural description then they would be treated as the same by the visual system, resulting in viewpoint-invariant recognition (Biederman & Cooper, 1991a, b). However, if the two views generate different structural descriptions (such as might happen if geons are obscured, or with rotations in the picture plane, where the spatial relations between geons are fundamentally changed) this would lead to a loss of viewpoint-invariance, or even an outright breakdown, of recognition.

In this paper, I review a range of findings that suggest that objects can be recognized in the absence of information pertaining to their orientation, which challenge the view that recognition is based on viewpoint-dependent representations in which shape and orientation are inextricably linked and processes that compensate for orientation differences. This evidence comes from dissociations between object recognition and processing of object orientation obtained from patients with neurological damage, as well as from healthy participants whose perception is placed under strain by having stimuli presented very briefly—thus, it is not a peculiar consequence of brain damage and/or neural reorganization. Collectively, these findings build a picture in which the initial step in object recognition is achieved through orientation-invariant means, possibly on the basis of component features, while coding the orientation of an object takes place after the initial recognition. Ultimately, both of these steps are required to deliver a veridical percept of an object in a specific orientation. While previous theories of object recognition by and large do not draw distinctions between conscious and unconscious processes in recognition, some of the findings reviewed here (especially in the section titled "Identity and Orientation Processing in Brief Displays") highlight important differences about the role that orientation plays in the recognition of attended and consciously identified objects vs objects recognized implicitly.

## Agnosia for object orientation: Object recognition without knowledge of object orientation

There are several reports in the literature of neurological patients who show a remarkable dissociation between a preserved ability to recognize objects presented in different orientations and a profound impairment in interpreting the orientation of these objects. This syndrome was first reported by the German ophthalmologist Friedrich Best in 1917 (Ferber & Karnath, 2002). Best described the case of a man (Z.) who had suffered extensive bilateral damage to the occipitoparietal lobes. Amongst other deficits of a spatial nature, Z. demonstrated a peculiar inability to determine the orientation of objects and pictures presented to him, even though he had no trouble recognizing what they were. For example, when shown letters, he could not discriminate between *n* and *u* and he could not determine which way a hand was pointing (up, down, left or right). Likewise, he could not tell whether a face was upright or upside-down, despite being able to identify the person in the photograph (apparently this was tested using a portrait of the Kaiser which, given other salient visual cues no doubt present in the picture, raises some questions about Z.’s real ability to recognize upside-down faces). Nevertheless, he was able to recognize upside-down objects easily.

Following this early case, the phenomenon of *agnosia for object orientation* was reported and studied more systematically in a number of other patients (Davidoff & Warrington, 1999; Fujinaga et al., 2005; Harris et al., 2001; Karnath et al., 2000; Martinaud et al., 2016; Robinson et al., 2011; Turnbull et al., 1995, 1997). Turnbull and his colleagues (1995, 1997) reported three cases (L.G., N.L., and S.C.), who showed a clear dissociation between their ability to recognize objects presented in different orientations in the picture plane (0°, ±90°, 180°) and their inability to interpret their orientation. For example, these patients could recognize an upside-down penguin but were as likely as not to say that it was correctly oriented. Further, when asked to match the orientation of pictures of objects presented in different orientations, L.G. was able to reorient a target picture to match the orientation of an upright model most of the time (indicating that she understood the task), but had great difficulty reorienting the target when the model was presented in an orientation other than the upright (Turnbull et al., 1995). Interestingly, she was able to match objects that did not have an intrinsic axis of orientation—such as arrows—almost perfectly, suggesting that her deficit was primarily in judging the orientation of objects that were misoriented relative to their usual canonical orientation. L.G.’s recognition was reported as being within normal limits, though it was not perfect, and often she took in excess of 10 s to name the object. Importantly, however, she was unable to determine the orientation of those objects that she had successfully recognized.

**Fig. 1:**
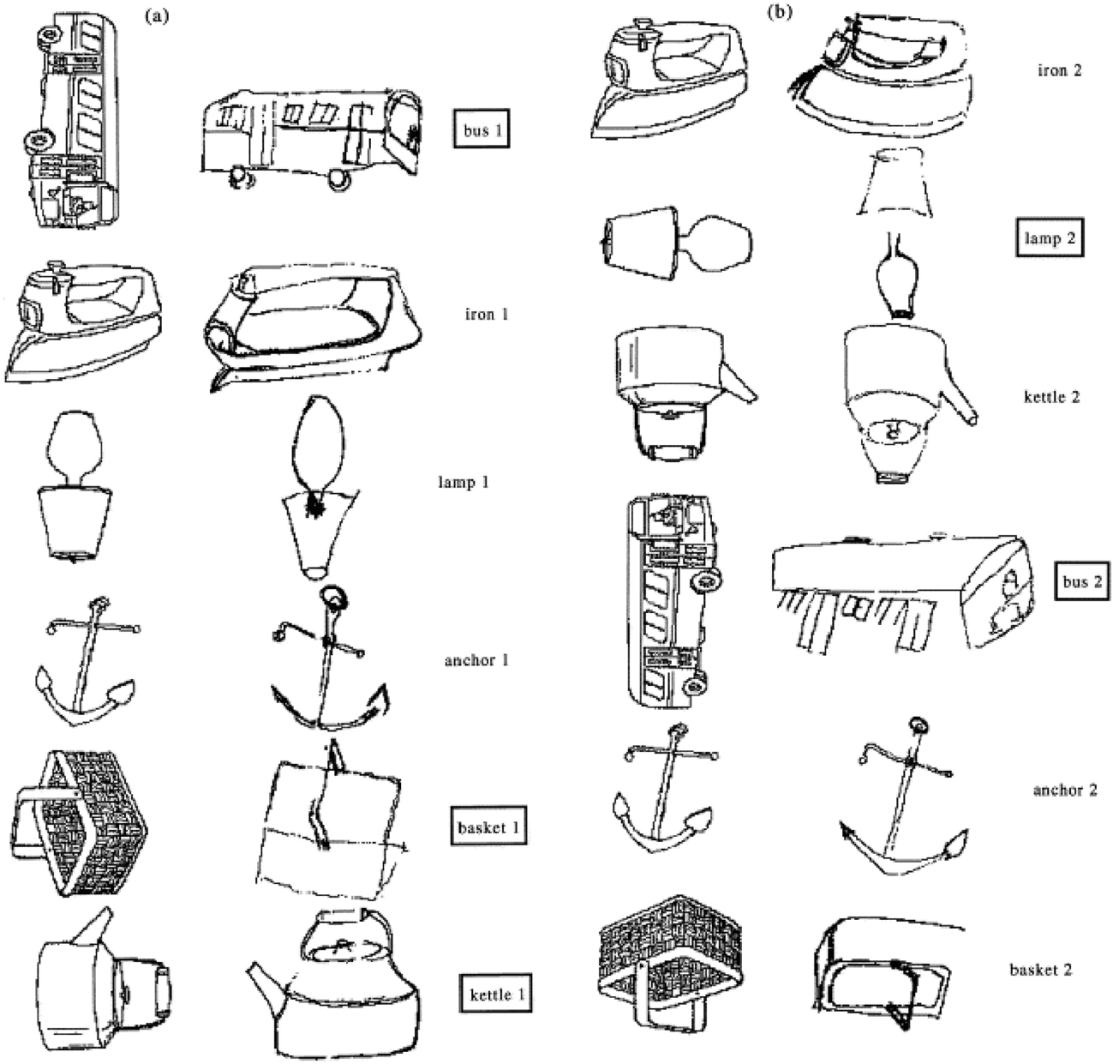
Copies of drawings produced by the orientation agnosic patient N.L. Orientation errors, all of which involve objects reproduced in the upright orientation, are highlighted. Reproduced with permission from Turnbull et al. (1997)

**Fig. 2:**
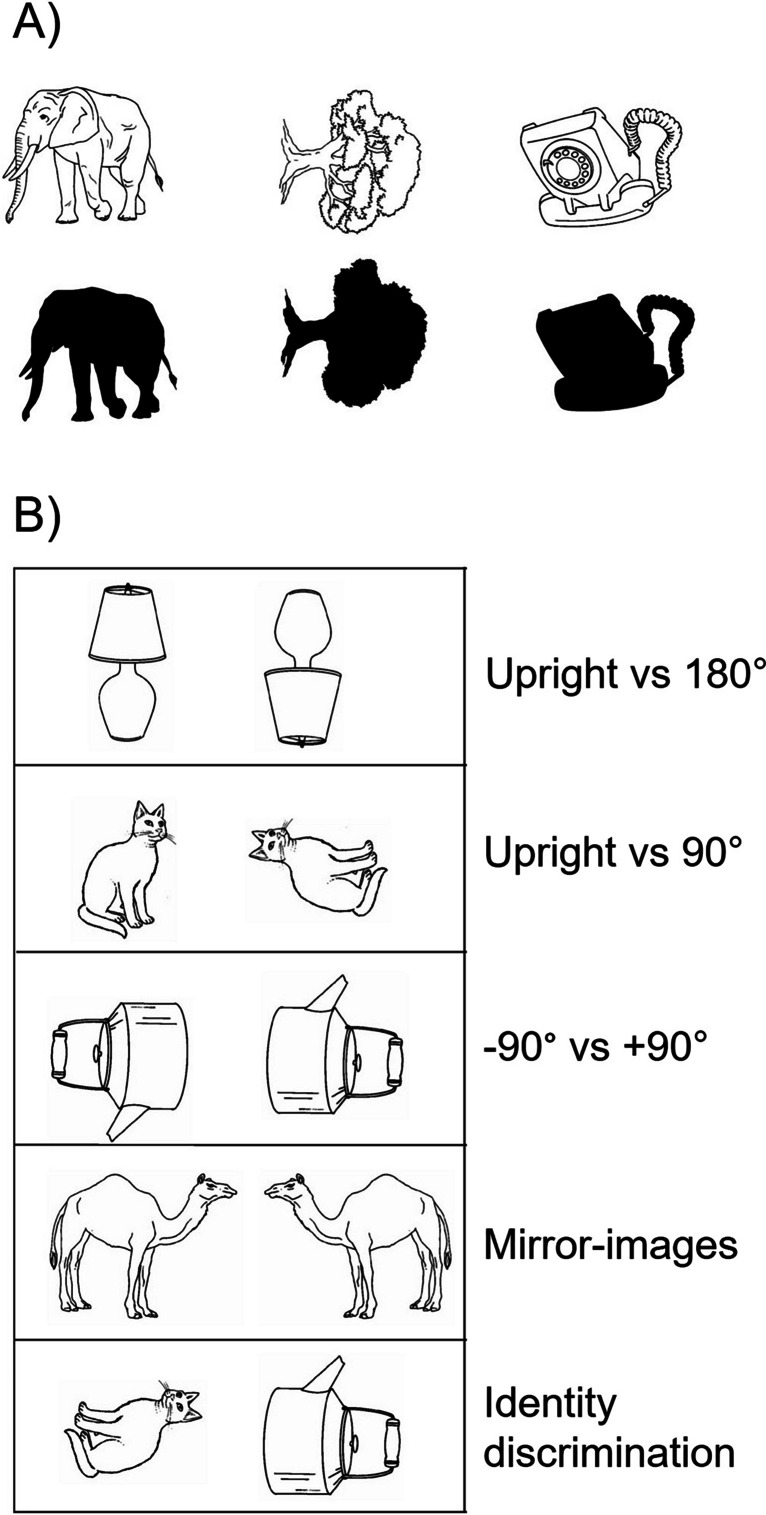
Examples of types of tasks commonly used to test knowledge of object orientation, adapted from Harris et al. (2001). **A** Patients are asked to name objects that are presented in different orientations or are asked whether the object is depicted in its correct canonical orientation. On some tests, they may be handed cards with the objects and asked to position them in the correct orientation. The figure shows examples of both standard line drawings and silhouette versions that were administered to E.L. (see text for details). **B** Examples of orientation discrimination tasks, as well as a control identity discrimination task. The patient is asked whether the two images are the same or different

A different patient, N.L., tended to reproduce misoriented drawings (e.g., a bus rotated by 90°) in their canonical orientation, again suggesting that he was unable to interpret the orientation of the misoriented model and that perhaps he was relying on an internal (upright) representation of the object to guide his drawing performance (Turnbull et al., 1997; see Fig. 1). In addition, N.L. had difficulty discriminating upright from upside-down drawings of identical objects, or mirror images of the same objects (see Fig. 2 for examples of such tasks). N.L. displayed more convincing evidence of intact object recognition, making no errors in object identification across a range of orientations. Further evidence consistent with orientation-invariant object recognition was obtained in a subsequent study in which N.L.’s reaction times were measured as he was naming misoriented objects (Turnbull et al., 2002). N.L. demonstrated a flat reaction time function across orientations, in contrast to control participants who showed the usual reaction time costs as the objects were rotated further from the upright (Jolicoeur, 1985; Jolicoeur & Milliken, 1989)—although his responses were slower overall than those of the control participants. The third case, S.C., showed a less pronounced deficit in judging the orientation of single objects, but he was unable to discriminate upright from upside-down pictures (Turnbull et al., 1997). His recognition abilities were also completely intact. All three patients described above also made orientation errors in spontaneous drawing and when copying drawings of objects or abstract figures.

Results obtained from another patient with orientation agnosia, E.L., suggested a possible explanation for the orientation-independent object recognition, based on the identification of salient features (Harris et al., 2001). Similarly to Turnbull et al.’s patients, E.L. displayed a profound impairment in judging the orientation of rotated objects and discriminating objects on the basis of orientation but had no difficulty recognizing line drawings of rotated objects, naming them quickly and with confidence in all orientations tested (±90°, 180°). However, E.L. was severely impaired when the objects were presented as black silhouettes with all the internal features obscured (see Fig. 2). In contrast, he could recognize upright silhouettes, indicating that the familiar contour of the object at this orientation was enough to enable successful recognition. This indicates that when the global shape was altered by rotating the object, recognition was only possible through the extraction of component features. At this stage, it is not known whether recognition by salient features is a strategy that was peculiar to E.L., or whether all orientation agnosics recognize rotated objects in this manner, given that none of the other patients were tested with silhouettes.

The patients described above represent perhaps the clearest examples of recognition of rotated objects in conjunction with a profound difficulty in interpreting the object’s orientation. Even though in some cases it could be argued that the patients’ recognition is not completely intact (e.g., L.G. sometimes took a long time to identify objects and was not always successful), the critical point is that in every case they had difficulty interpreting the orientation of objects that they *could recognize successfully*. In other words, their recognition was achieved on the basis of orientation-independent representations. This poses significant challenges to viewpoint-dependent theories of recognition which argue that orientation is an integral part of the representations and processes that underlie recognition. Turnbull and colleagues (1997) had suggested that these findings provide evidence for the existence of an orientation-independent route to object recognition, implemented in the ventral visual stream, in the absence of an orientation-dependent representation mediated by the dorsal visual stream. This suggestion is supported by the fact that most patients with this syndrome have documented damage to areas of the dorsal stream encroaching on the parieto-occipital regions, with the area of most overlap being the right parietal lobe (Harris et al., 2001; Karnath et al., 2000; Martinaud et al., 2016; Turnbull et al., 1995, 1997).

A final case that warrants a mention is a recently reported case identified as Davida, described by Vannuscorps et al. (2022). This fascinating case has no known brain lesion but presents with a very selective deficit in determining the orientation of high-contrast visual stimuli, arguably processed in the parvocellular pathway. Davida has no difficulty whatsoever recognizing objects and shapes, but her performance is marked by orientation confusions both in perception and in action, including familiar objects, shapes, letters identified by their orientation (e.g., *b*, *q*). One thing that distinguishes Davida from other orientation agnosic patients is that she experiences objects as rapidly changing in orientation as she looks at them (whereas the other patients typically state that the objects “look right” even if they are misoriented, or sometimes claim that the object does not have an actual upright orientation). Although Davida does not completely fit the picture of orientation agnosia described above, she nevertheless demonstrates a dissociation between intact recognition and aberrant orientation processing, also suggestive of recognition via viewpoint-independent means. Vannuscorps et al. (2022) speculated that Davida was able to generate shape-centered representations at an early stage of visual processing that is common to the ventral and dorsal visual streams but had a deficit in mapping these shape-centered representations onto spatial reference frames that enable conscious perception and guide actions, implemented in the dorsal visual stream (Goodale & Milner, 1992).

In sum, these diverse cases present convincing evidence that object recognition is possible in the absence of representations and processes that code the orientation of objects, which goes against the predictions of viewpoint-dependent theories of object recognition. While it might be possible to argue that these patients’ preserved recognition is supported by early, viewpoint-dependent (i.e., in a retinotopic reference frame) representations, the reported findings do not provide any hints of orientation sensitivity in either their accuracy, or their reaction times (where those were measured; Turnbull et al., 2002), making this possibility unlikely. It is important to stress that the evidence indicates that object orientation has little effect on these patients’ *recognition*. As I will describe in the next section, there is equally clear evidence that most of these patients do demonstrate some sensitivity to orientation and this sensitivity manifests itself in their ability to *interpret the orientation of the objects*.

## Orientation processing in agnosia for object orientation

As outlined in the previous section, a consistent finding in patients with orientation agnosia is their generally well-preserved object recognition, including for misoriented objects. However, while they all exhibit difficulties in determining object orientation, there is considerable heterogeneity in these deficits. For example, as mentioned earlier, some patients seem to have residual knowledge of the usual upright orientation of objects. Patient L.G. was better able to match the orientation of objects when the model was upright, and patient N.L. tended to reproduce misoriented drawings in an upright orientation (Turnbull et al., 1995, 1997). This was even clearer in two other patients, E.L. (Harris et al., 2001) and K.B. (Karnath et al., 2000), who did not make any errors in their orientation judgments when the objects were upright and were also much more likely to place the object upright than in other orientations, when asked to demonstrate the canonical orientation of misoriented objects. Indeed, Karnath and colleagues (2000) questioned whether the label “object orientation agnosia” was warranted in K.B.’s case, given that she seemed to have preserved knowledge of the correct orientation (I believe the label is warranted for E.L., because he had difficulties with orientation discrimination tasks. Note that K.B. was not tested on such tasks, which may mask a more pervasive orientation perception disorder, as well as the fact that she had difficulty reorienting letters to demonstrate the correct letter orientation which also speaks to a broader deficit in interpreting orientation). Nevertheless, such findings do suggest that there is at least some residual knowledge of the correct canonical orientation of objects in most of these patients that can help guide their performance when an object matches that orientation.

In addition, E.L. was also significantly more accurate in judging the orientation of upside-down objects than of objects rotated by 90° (Harris et al., 2001). He was also able to discriminate between an upright and an (identical) upside-down object, but his discrimination of other orientations—such as the upright versus a 90° rotation, or an object rotated by 90° clockwise and the same object rotated by 90° counterclockwise—was at chance (see Fig. 2 for examples of the tasks). Thus, in addition to recognizing the correct upright orientation, E.L. also demonstrated some facility in processing the orientation of objects rotated by 180°. Notably, this only applied to objects with an intrinsic canonical upright orientation; he was not able to discriminate the orientation of arrows pointing up and down. Patient K.B., reported by Karnath et al. (2000), was also better at judging the orientation of inverted objects than of objects rotated by 90° and when asked to demonstrate the correct orientation of wooden letters, she apparently made some errors by placing the letters at 90° or mirror-reversed, but never upside-down. Along similar lines, although Turnbull et al.’s patient N.L. tended to reproduce misoriented drawings in an upright orientation, he only did this for models presented at 90° from the upright; he copied upside-down objects correctly in an upside-down orientation (see Fig. 1)—although, unlike E.L., he had difficulties on upright-upside-down discriminations and was noted to have a peculiar preference to hang pictures upside-down in his room. A potential interpretation of E.L.’s relative facility with upside-down objects offered by Harris et al. (2001) is that it is easier to interpret the orientation of an object when its principal axis is aligned with that of a representation of the object stored in memory (in its canonical orientation), as is the case when the object is depicted upright or rotated by 180°. In contrast, other orientations are more difficult to interpret, because the axis of the object has to be located and compared with that of the stored representation—a process known as establishing axis correspondence, that underpins the mapping of an object-centered representation onto an external reference frame (McCloskey et al., 2006). It is worth noting that a similar pattern has been found in neurologically normal participants, who are also more accurate when judging the orientation of briefly presented upright and upside-down objects, compared with objects rotated by 90° (De Caro, 1998; Harris & Dux, 2005a, Exp. 3; Harris et al., 2020). Taken together, these findings suggest that the memory representation of an object contains information about the canonical orientation of that object (when that exists) and this information can facilitate the interpretation of orientations, such as upright and 180°, where the principal axis aligns with that of the stored representations.

However, it should be acknowledged that other cases do not show this selective facility with upright and inverted objects, presenting instead with either a pervasive orientation impairment that affects all orientations, or with milder deficits confined only to small deviations from the upright orientation that do not encompass the 90° cardinal orientation (Cooper & Humphreys, 2000; Fujinaga et al., 2005). Finally, two cases showed a more profound difficulty with 0° and 180° than 90° orientations (Robinson et al., 2011; Vannuscorps et al., 2022). It may be worth noting that these two cases are ones who do not have a demonstrable lesion on brain scans. The case reported by Robinson et al. (2011) suffered from a complex regional pain syndrome, which the researchers speculate may have led to aberrant reorganization of cortical networks, and the case reported by Vannuscorps et al. (2022) had no known pathology (but potentially also aberrant cortical organization). This last case (Davida), in particular, has the unusual experience that objects rapidly change their orientations as she is looking at them. Intriguingly, she is in fact least likely to settle on the actual depicted orientation when selecting what orientation to report.

The analysis of these deficits suggests that the main difficulty experienced by patients with agnosia for object orientation is in mapping the orientation of the object they are looking at to that of an internal representation, that is, a problem of establishing the axis correspondence between a viewer-centered and an object-centered reference frame.

## The special case of mirror images

The ability to discriminate between mirror images is a special instance of orientation discrimination because it represents a reflection (i.e., a change in axis polarity), rather than a change in axis orientation. Some of the patients with agnosia for object orientation, though not all, had additional difficulties discriminating left-right mirror images (Davidoff & Warrington, 1999; Harris et al., 2001; Turnbull et al., 1995, 1997; Vinckier et al., 2006). There are also cases who had a selective deficit in discriminating mirror-images, without an agnosia for object orientation as defined above (Davidoff & Warrington, 2001; Martinaud et al., 2014; Priftis et al., 2003; Turnbull & McCarthy, 1996). This double dissociation between discrimination of mirror images and other orientations suggests that these are subserved by different mechanisms, an idea supported by a recent voxel-lesion symptom mapping study which investigated the neural bases of mirror-image versus other orientation perception deficits in a larger group of patients with parietal lesions and found that these two deficits indeed mapped to distinct subregions of the posterior superior temporal gyrus and inferior parietal lobules (Martinaud et al., 2016).

Mirror-image confusion—especially for left-right mirror-images—is also quite pervasive during childhood. Children before about the age of seven tend to confuse reflected letters such as *b* and *d* and often produce mirror-writing as they learn to read and write, and some people continue to exhibit such difficulties all their lives (Corballis & Beale, 1976; Gregory et al., 2011; McCloskey, 2009). People are also notoriously poor at recalling the left–right orientation of even very familiar pictures and scenes (Kosslyn & Rabin, 1999), and other species—including octopuses, dogs, and monkeys—also find it difficult to tell lateral mirror images apart (Corballis & Beale, 1976; Walsh, 1996). It has been suggested that mirror-image “confusion” is actually an adaptive mode of processing visual information because most often mirror images represent two sides of the same object and, therefore, it would be expedient for the visual system to treat them as identical (Walsh, 1996). This idea is supported by an intriguing case reported by Warrington and Davidoff (2000), whose mirror-image discrimination was impaired for objects that she recognized successfully, but who could discriminate between mirror-images of objects that she failed to recognize. Monkeys with lesions to the inferior temporal cortex provide related evidence: These lesions result in recognition impairments, but they are accompanied by a relative improvement in the ability to discriminate mirror images (recall that monkeys are generally poor at this), as if the monkey were “released” from the tendency to generalize across mirror-images (Gross, 1978). The fact that mirror-image discrimination is effortful and must be learnt is telling, as it is further evidence that perception of orientation is a process that occurs independently of recognizing the object, just as findings from the patients with orientation agnosia suggest. In the following section, I review evidence from studies that strain perception by using very brief stimulus exposure that also supports this notion.

## Identity and orientation processing in brief displays

Some studies have probed the nature of the representations coded at the very early stages of visual processing in neurologically normal participants by presenting object stimuli very briefly. Initial findings indicated that participants can verify the identity of objects faster than their orientation when presented with brief, backward-masked pictures of objects or alphanumeric characters in different orientations (Corballis et al., 1978; De Caro, 1998; De Caro & Reeves, 2000). In these tasks, participants see a description, such as “UPRIGHT CAR” or “ROTATED TREE,” which is followed by a stimulus which matches or does not match the description in identity, orientation, or both; the participants answer “yes” if the picture fits the description in its entirety and “no” otherwise. Thus, it is possible to analyze the effects on performance of an identity or an orientation mismatch and deduce whether identity or orientation is processed earlier. These studies all indicate that identity is processed before orientation, because identity mismatches are detected from briefer exposures than orientation mismatches. Furthermore, De Caro and Reeves (2000) found that the time taken to verify object identity was not affected by the degree of misorientation—except that it was generally slower to identify misoriented objects than upright objects—whereas the time taken to verify the object’s orientation increased systematically as a function of the object’s misorientation from the canonical upright. This led them to suggest that the orientation effects seen in naming tasks are due to a process of determining the object’s orientation, which occurs after recognition, in line with the theoretical proposal put forward by Corballis (1988), who argued that the viewpoint-dependent effects on naming are due to a process of establishing the orientation of an object and refining the initial crude viewpoint-invariant recognition.

**Fig. 3:**
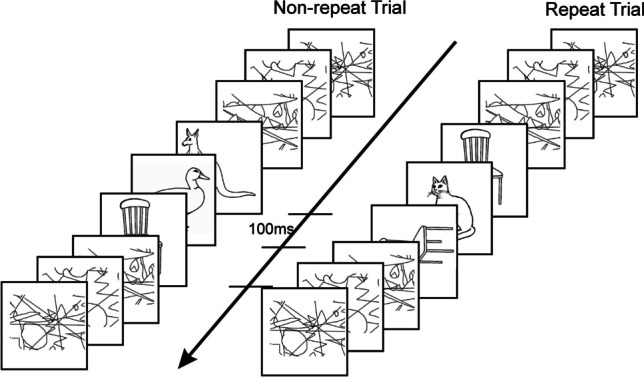
Illustration of the experimental paradigm used in the repetition blindness studies with rotated objects. Stimuli were presented for 100 ms each, with no blank interstimulus interval and participants had to name the objects (NB. on some catch trials there are only two objects). The critical items are the first and third pictures in the stream, which were either the same object (repeat trials) or different objects (non-repeat trials), presented in either the same orientation (both upright, left stream) or in different orientations (right stream)

Converging evidence emerged from studies that used rapid serial visual presentation (RSVP) of objects. In RSVP, visual stimuli are presented sequentially in the same spatial location at a rate of 8–12 items/s and the participants are required to report either a subset of items (e.g., one or two targets defined by a specific color or category) while ignoring the others, or all the items (usually in shorter streams that do not exceed working memory capacity). Several limitations of temporal attention and perceptual awareness have been demonstrated using this technique, and the study of these phenomena provides important insights into the early stages of visual cognition, as well as the processes by which a stimulus is consciously perceived. One phenomenon that occurs under these conditions is *repetition blindness* (RB)—a failure to detect and report both occurrences of a repeated item when they appear in close temporal proximity (usually up to 400–500ms; see Fig. 3 for a depiction of the task). This failure to report a repeated item is not due to masking or the limits of RSVP processing, because if a *different* item is presented at the same serial position, it is usually detected and recalled successfully. Thus, RB can be taken as a measure of implicit recognition because the repetition of the item must be registered at some level of processing, although it fails to be consciously perceived as a distinct visual occurrence. RB has been attributed to a difficulty in establishing separate *tokens,* or episodic instances, of the same *type* representation (Kanwisher, 1987).

Several studies have used RB to probe the nature of the type representations used in object identification. The logic here is that if objects are represented in a viewpoint-invariant fashion, RB should occur between identical objects presented in different orientations because these stimuli would converge on the same type representation. Conversely, if objects are presented in viewpoint-dependent fashion, then RB should not occur between repeated objects presented in different orientations, because these would activate distinct type representations. Moreover, if rotated objects need to be normalized through rotation, then one should see a systematic modulation of the RB as the orientation difference between repeated objects increases. We and others (Harris & Dux, 2005a, 2005b; Hayward et al., 2010; Kanwisher et al., 1999) have found that RB occurs when the repeated objects are in different orientations and that the magnitude of the RB deficit does not vary systematically as a function of the orientation difference between the critical items, in line with the idea that the activation of type representations is orientation invariant. Corballis and Armstrong (2007) found similar results using orientation-sensitive letters that have the same shape such as *p*, *q*, *b*, and *d*, with all combinations of such letters resulting in RB. There is some evidence from these experiments that the recognition indexed by RB might be achieved on the basis of component features, because RB also occurs between pictures of the same item that have been cut up and rearranged in different configurations (Hayward et al., 2010, Exp. 5).

Interestingly, some of these experiments have also shown that RB is diminished and sometimes completely abolished, when the critical items are objects with a canonical upright orientation presented in the upright (0°) and 180° orientations (Harris & Dux, 2005a, 2005b; Hayward et al., 2010). This finding echoes the facility in interpreting these orientations demonstrated by some patients with orientation agnosia and suggests that in this case observers can more efficiently form object tokens and avoid repetition blindness. In contrast, for objects presented in other orientations that are harder to interpret (e.g., 90°), the little time available under RSVP conditions might not be sufficient to enable participants to extract sufficient orientation information to determine that it is the same object presented in a different orientation, leading to a failure to create a separate spatiotemporal token and resulting in RB (Harris & Dux, 2005a). Taken together, the object verification and the RB findings reviewed here led to the hypothesis that the initial activation of object types, or identity representations, is orientation-invariant but that coding of the objects’ orientation may be critical in establishing a conscious representation that enables report. Support for this hypothesis comes from studies that employed the attentional blink, which I turn to next.

**Fig. 4:**
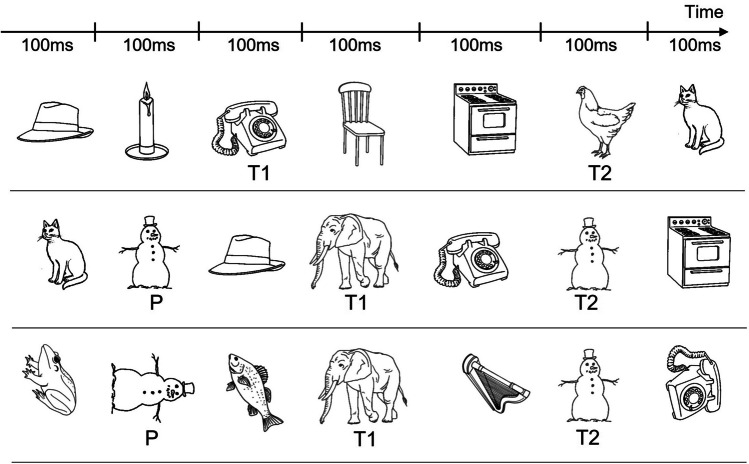
Examples of RSVP streams used in attentional blink experiments with objects. The streams typically consist of 10–12 items presented for 100 ms each in the same spatial location (the above shows a subset of the stream). Two red targets, depicted here as T1 (Target 1) and T2 (Target 2), are presented amongst black distractors, separated by varying lags; the participants must report these targets. Top row: An example of a ‘standard’ trial consisting of all upright objects, with the two targets separated by a lag of 3 items. Middle row: An example of a trial containing a priming distractor (P), which is the same object as T2 and in this case is in the same orientation as T2. In this example, T1 and T2 are separated by a lag of 2 items. Bottom row: An example of a trial containing upright targets and rotated distractors, including a priming distractor that differs in orientation from T2. See text for further details

Another limitation of temporal attention somewhat similar to RB is the *attentional blink *(AB), an impaired ability to report the second of two target items (which are not identical), if they appear within ~600 ms of one another in a RSVP stream (Raymond et al., 1992). In a typical AB experiment, observers are required to monitor a stream of visual stimuli for the presence of two targets (Target 1 and Target 2), usually denoted by a different color or category, while ignoring distractor items (see Fig. 4). It is well-established that all items in a RSVP stream, be they targets or distractors, are initially recognized to conceptual levels (Potter, 1976, 2012) but have to undergo further processing if they are to be consolidated into a reportable form (Chun & Potter, 1995; Jolicoeur, 1999). This additional processing is held to be responsible for the AB, as items in close temporal proximity to Target 1 must wait to be consolidated and are, therefore, more susceptible to decay and interference from other items. Previous studies have shown that the magnitude of the AB is sensitive to variables that affect attentional processing between the initial registration of stimuli and their subsequent consolidation into a more durable form, such that a more attentionally demanding Target 1 task can lead to a bigger blink (Olson et al., 2001). Therefore, the AB paradigm offers a very useful context in which to investigate the effects of orientation on processing objects that are the focus of attention and get consolidated (targets) compared with objects that do not received attention and might not reach conscious awareness (distractors and items affected by the AB).

Using such manipulations, Dux and Harris (2007b) found that if Target 1 was an object rotated by 90°, this produced a larger AB than when this object was upright or rotated by 180°. This is consistent with findings reviewed above that 0° and 180° orientations are processed more efficiently than 90° orientations. In contrast, the magnitude of the AB was not affected by the orientation of the distractors (all upright compared with a mix of rotated orientations) if the targets themselves were upright. Crucially, this was also the case when the targets were defined by category (animals amongst nonanimal distractors), meaning that the distractors were sufficiently processed to activate their semantic category (Dux & Harris, 2007b). In further experiments, Harris et al. (2010) also found that when the same object as Target 2 was included earlier in the stream as a distractor (colored black, as opposed to the red targets that the observers were searching for; see the lower two panels of Fig. 4) this primed report of this target (i.e., reduced the magnitude of the AB). This priming was modulated both by the orientation of the distractor and the amount of attention it received. If the priming distractor was placed after Target 1 during the time window of the AB, it primed Target 2 equally when it was in the same orientation as the target and in a different orientation. However, if the priming distractor occurred outside the AB window, it induced positive priming of Target 2 only if it was in a different orientation from the target, but no priming or even negative priming if it was in the same orientation as the target. This may seem counterintuitive at first, but we know that distractors are actively inhibited during RSVP, as the observers attempt to selectively attend to targets (Dux et al., 2006; Olivers & Meeter, 2008; Olivers & Watson, 2006), and this inhibition fails during the AB window, presumably because it requires attention and cognitive control resources (Dux & Harris, 2007a). Thus, these findings indicate that orientation-invariant identity information is extracted from all items, even in the absence of conscious awareness (i.e., during the AB), but that for attended items (be they targets or distractors that are not affected by the AB) additional information about the object’s orientation is integrated in the object representation. In the case of targets, this additional step makes it harder to recognize Target 1 when it is rotated than when it is in the usual upright orientation (or upside-down), leading to a more profound AB for subsequent items. In the case of distractors, the suppression seems to affect that specific orientation-dependent representation, leaving priming on the basis of other orientation-invariant features intact. While it is possible that this priming is mediated by relatively low-level perceptual features, it is more likely that it occurs on the basis of a higher-level identity representation, given demonstrations of conceptual processing of items in RSVP streams (Potter, 1976, 2012) and of semantic priming from distractors subject to the attentional blink (Harris & Little, 2010; Maki et al., 1997).

A final piece of evidence comes from a study by Harris et al. (2008) in which participants had to name an upright object that was preceded by a brief masked prime presented in different orientations, whose identity either matched or did not match the target object, and which participants were instructed to ignore. The duration of the prime varied from 16 ms to 350 ms. Significant priming (i.e., faster naming of the target object when preceded by the same object prime) was found for primes longer than 70 ms, and this priming did not vary as a function of the prime orientation. In a separate experiment, participants saw the same rotated primes for extended time and had to name them; here, the naming times increased systematically with the degree of rotation from the upright, as has been found in numerous studies (e.g., Hamm & McMullen, 1998; Jolicoeur, 1985; Jolicoeur & Milliken, 1989; Tarr & Pinker, 1989). We took this as confirmation that the orientation effects on naming are incurred at a later stage when the object’s representation is consolidated in visual short-term memory for report (Corballis, 1988; De Caro & Reeves, 2000; Dux & Harris, 2007b).

Taken together, the results from studies reviewed in this section demonstrate that initial recognition of items in brief displays is orientation-invariant and occurs even in the absence of conscious awareness, and that orientation information is integrated in the object’s representation at a later stage of processing, when items are selectively attended and consolidated in a reportable form.

## Object identity and orientation as separate features that require binding

The research reviewed so far points to independent coding of object identity and object orientation and suggests that interpreting the orientation of an object is a process that happens after the initial recognition of its identity—or perhaps it never happens, as in the case of patients with agnosia for object orientation. That said, in the majority of the studies with healthy participants, the effects of orientation were measured indirectly, by looking at how the orientation of items modulates recognition. In a departure from this, Harris et al. (2020) tested explicit knowledge of both identity and orientation of a probed item. Participants viewed a two-item RSVP stream, preceded and followed by a mask, with each of these items shown for 70 ms. The two items were line drawings of common objects with a canonical upright orientation and were presented in a variety of orientations (90° clockwise or counterclockwise or 180° from the upright; always different for the two objects). This was followed by an object presented upright and the participant had to indicate whether this object had appeared in the previous stream or not, and then had to indicate its orientation by using the arrow keys on the keyboard, guessing if necessary. They were required to respond with an orientation even if the object had not been part of the stream (i.e., it was a recognition foil) in order to measure any biases in responding.

Several interesting findings emerged. First, while recognition of the item (as measured by *d′* sensitivity) was quite high, participants only reported the correct orientation of about 70% of the items they had correctly recognized—and in accord with other findings described above, they were more accurate in judging the orientation of objects rotated by 180° than objects rotated by 90°. Second, when they failed to identify the object correctly, their orientation judgement was no better than chance. Together, these results indicate that object identity is processed before its orientation, and knowing the identity is a *necessary* step for determining the object’s orientation. Third, when participants reported an incorrect orientation, they were much more likely to report the orientation of the other object present in the stream than an orientation that had not be presented—in other words, their errors tended to be incorrect bindings of the identity and orientation of the two objects (see also Corballis et al., 2007, for similar findings using letter stimuli). Interestingly, this propensity for binding errors was only observed when the two objects were presented sequentially in the same spatial location, but not in other experiments where the two objects appeared at different spatial locations (Harris et al., 2020). A similar pattern was also reported by Pertzov and Hussain (2014) using sequences of lines of different orientations and colors. When their participants were cued to report the orientation of a line of a specific color, they made binding errors by reporting the orientation of another line presented at the same location but not the orientation of a line presented at a different location. This suggests that spatial location might help to maintain the correct bindings between object features (Treisman & Gelade, 1980; Treisman & Zhang, 2006).

## Putting orientation in the picture

The research reviewed above highlights a number of important conclusions. The first one is that orientation is not a defining feature of the object representations that mediate recognition. This is clearly demonstrated by the findings from patients with agnosia for object orientation, who can recognize objects without understanding their orientations, as well as by the fact that the initial activation of object representations from brief displays is largely orientation invariant.

The second conclusion is that the effects of misorientation often seen in object recognition tasks appear to reflect processes involved in determining object orientation rather than object identity. This is demonstrated by the fact that any sensitivity to orientation displayed by patients with agnosia for object orientation is evident in orientation judgement tasks, rather than in recognition tasks; as well as by the fact that the speed of verifying the orientation of objects in brief displays is affected by orientation.

The third conclusion is that although orientation is not a defining feature of the object representation that mediates initial recognition, orientation information is nevertheless subsequently integrated in the representations of objects that we attend to and are conscious of (as well as those that guide our actions, although this aspect is outside the scope of this review). This is demonstrated by the fact that attended targets show systematic orientation effects in the speed with which they are processed and named. In fact, I would argue that resolving the object’s orientation is a crucial step in establishing a conscious percept of an object. When orientation processing falters, the object is generally still perceived as being in *some* orientation. Sometimes this might be the orientation of a different object occurring in close temporal proximity, or the canonical orientation (e.g., for patients with orientation agnosia who presumably default to the orientation of their memory representation of the object), meaning that when objects are consciously experienced, they are always experienced in a particular orientation—which may or may not be the veridical one. A potential exception to this might be Davida, the case reported by Vannuscorps et al. (2022), whose perception was one of the object not having a stable orientation, but alternating between different orientations. Davida is unusual in that her orientation deficit was confined to stimuli with particular characteristics suggestive of dysfunction specific to the parvocellular pathway. At the same time, her deficit is likely developmental in nature, as there was no obvious pathology that could explain her symptoms and, therefore, her case may not be representative of the normal way in which orientation is processed. At this stage it is not clear how to interpret her phenomenal experience—although it has been suggested that it might indicate a failure to maintain stable bindings between a shape and its orientation (Harris, 2022; Vannuscorps et al., 2022).

Determining the orientation of objects appears to require that an object-centered representation be mapped onto an external spatial reference frame. This is a hypothesis that was entertained by Corballis (1988), who suggested on logical grounds that an object must be recognized before one could determine where its top was and, therefore, whether it was positioned in its expected canonical orientation or some other orientation. Corballis believed that a process of double-checking the orientation was responsible for the orientation-dependent effects on recognition. The findings reviewed here provide empirical support for this intuition.

McCloskey and his colleagues (McCloskey, 2009; McCloskey et al., 2006) articulated a formal conceptualization of object orientation as a relationship between an internal reference frame (centered on the object, which specifies the location of component features relative to the principal axis of the object) and an external reference frame that could be either an egocentric frame based on the observer, or some environmental frame of reference, such as the direction of gravity, the walls of a room or the edges of a screen. According to this theory, determining an object’s orientation involves (1) establishing axis correspondence between the internal and external frames of reference (i.e., deciding which internal axis corresponds to which external axis), (2) establishing axis polarity (whether the pole of the internal axis aligns with the pole of the external axis, or whether they have negative polarity, i.e., they are reflected relative to each other), and (3) calculating axis tilt (the angular displacement between the corresponding axes). Difficulties in any of these steps can result in specific types of orientation errors. The inability to interpret object orientation, as seen in patients with agnosia for object orientation represents a failure to establish axis correspondence (Harris et al., 2001), while errors in making more difficult orientation discriminations, such as amongst oblique orientations, represent failures to establish axis tilt. A failure to establish axis polarity would result in mirror-image confusions, as well as certain spatial transposition errors (Gregory et al., 2011; McCloskey et al., 2006).

The binding errors between objects and orientations described in the previous section (Harris et al., 2020; Pertzov & Husain, 2014) suggest that once a representation of orientation is established, this is integrated with the object’s representation retrieved from memory at the time of recognition, and that this binding has to be actively maintained, otherwise these features may become “unbound,” giving rise to misbindings of identity and orientation (Corballis et al., 2007; Harris et al., 2020). This would also explain why priming from an object that is subject to an attentional blink generalizes across orientations (Harris et al., 2010)—because it occurs on the basis of a representation that is not tied to any particular orientation, due to the lack of attention that is required to maintain the binding between object identity and its orientation.

## Conclusion

The neuropsychological investigations of patients with deficits in orientation processing, together with the findings of experimental tasks that challenge perception in healthy observers reviewed here provide converging evidence that object orientation is not an inherent property of the object representations that subserve object recognition. The initial recognition appears to be achieved on the basis of orientation-invariant representations, largely in the absence of attention and conscious awareness, while information about the orientation is incorporated at a later stage when the activated object representation is mapped to an external spatial reference frame. This additional step results in a conscious representation of the object in a particular orientation, but this step may fail after damage to brain areas that implement spatial transformations, as in patients with orientation agnosia, or when insufficient time is available to achieve the spatial mapping, leading to binding errors between the identity and orientation of objects.
